# Regional Anesthesia in a Cirrhotic Patient With Coagulopathy and Pulmonary Hypertension: A Case Report

**DOI:** 10.7759/cureus.91035

**Published:** 2025-08-26

**Authors:** Angela Lu, Katherine Elsea, David A Olsen, Beth A VanderWielen

**Affiliations:** 1 Anesthesiology and Perioperative Medicine, Mayo Clinic, Rochester, USA

**Keywords:** axillary brachial plexus nerve block, cirrhosis, coagulopathy, regional vs. general anesthesia, ultrasound-guided regional anesthesia, upper limb orthopedic surgery

## Abstract

End-stage liver disease increases perioperative risk due to cirrhosis-related complications such as portal hypertension, coagulopathy, ascites, encephalopathy, and impaired anesthetic metabolism. Although regional anesthesia may reduce these risks compared to general anesthesia, its use is often limited in this population due to coagulopathy and altered drug metabolism. We present the successful use of an axillary brachial plexus block with bupivacaine for olecranon bursa excision in a 72-year-old man with ASA (American Society of Anesthesiologists) IV physical status classification and a MELD-Na (Model for End-Stage Liver Disease-Sodium) score of 15, complicated by coagulopathy, portal hypertension, and pulmonary hypertension. This case highlights the feasibility of regional anesthesia in high-risk cirrhotic patients with abnormal coagulation profiles.

## Introduction

Anesthetizing patients with hepatic dysfunction poses significant challenges. Severe hepatic dysfunction increases the risk of perioperative morbidity and mortality, including hypoxia, hypotension, drug-induced organ decompensation, delayed emergence from general anesthesia, and postoperative encephalopathy. Patients with cirrhosis often experience longer hospital stays, increased transfusion requirements, and postoperative complications such as bleeding, encephalopathy, infection, and heart failure [1-6].

Progressive hepatic disease affects multiple organ systems, including pulmonary, renal, and cardiovascular systems. It is associated with peripheral and splanchnic vasodilation and a reduced vascular response to vasoconstrictive stimuli. As hepatic dysfunction advances, vasodilation becomes more pronounced, contributing to a hyperdynamic circulatory state and renal artery vasoconstriction. These systemic hemodynamic alterations can impair cardiac and renal function, elevating perioperative risk [7,8]. Pulmonary hypertension further increases perioperative morbidity due to elevated right ventricular strain and reduced preload [2,7,9]. Regional anesthesia may help mitigate these risks by minimizing hemodynamic fluctuations [9].

Postoperative cognitive dysfunction is common in cirrhosis, and distinguishing it from hepatic encephalopathy can be challenging [4]. Regional anesthesia may reduce this risk by limiting systemic pharmacologic exposure [2]. However, coagulopathy and thrombocytopenia often limit the feasibility of regional techniques [2,9]. Conventional coagulation tests may not reliably predict bleeding risk in cirrhotic patients [10].

There is limited literature evaluating the safety of regional anesthesia in cirrhotic patients with coagulopathy. We present the case of a patient with cirrhosis, thrombocytopenia, portal hypertension, and pulmonary hypertension who underwent olecranon bursa excision under axillary brachial plexus block. This case was institutional review board (IRB) exempt, with informed consent obtained.

This case was previously presented at the 2025 American Society of Regional Anesthesia meeting.

## Case presentation

**Figure 1:**
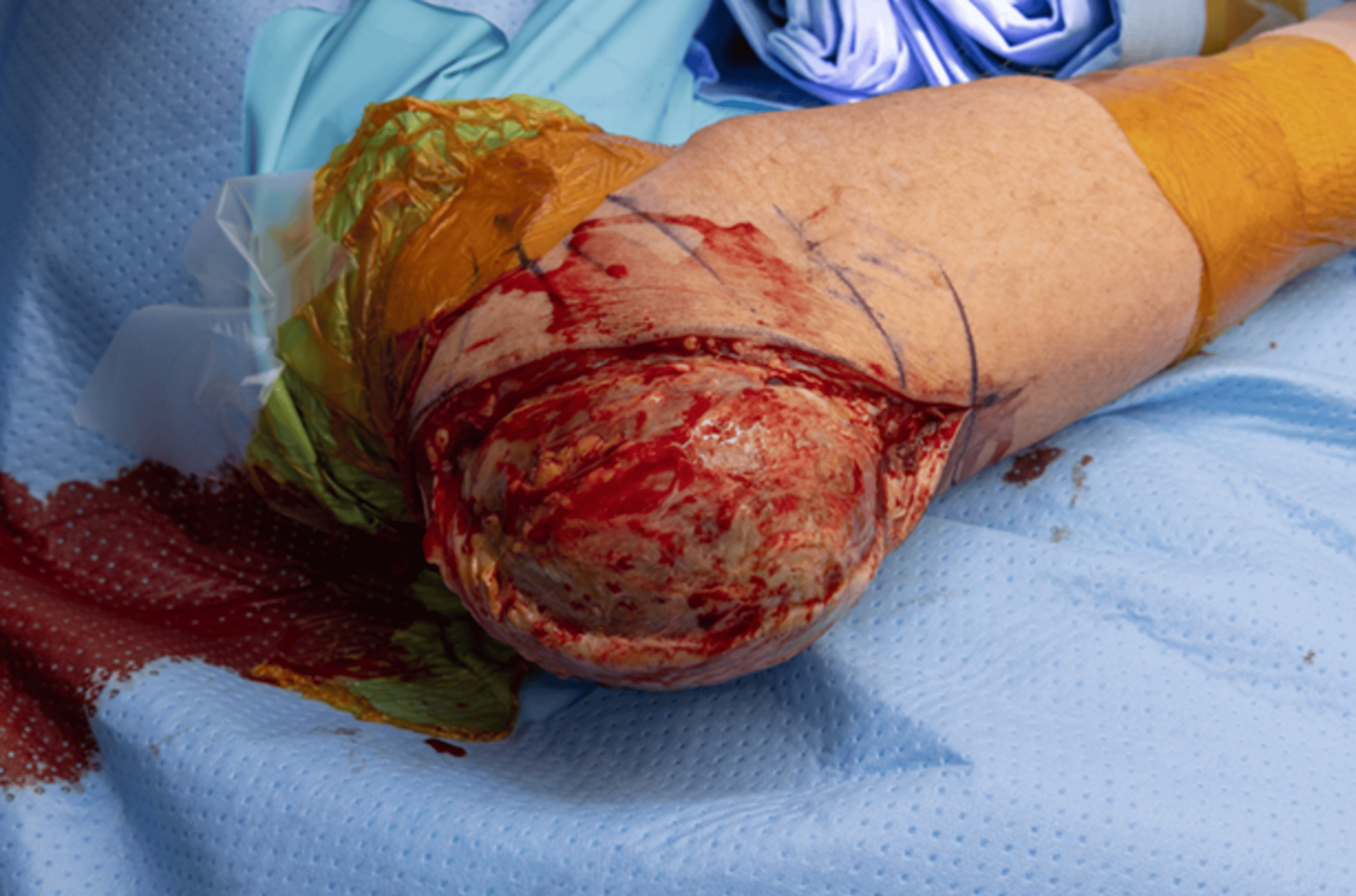
Photo after the surgical incision of right elbow olecranon bursitis

**Figure 2:**
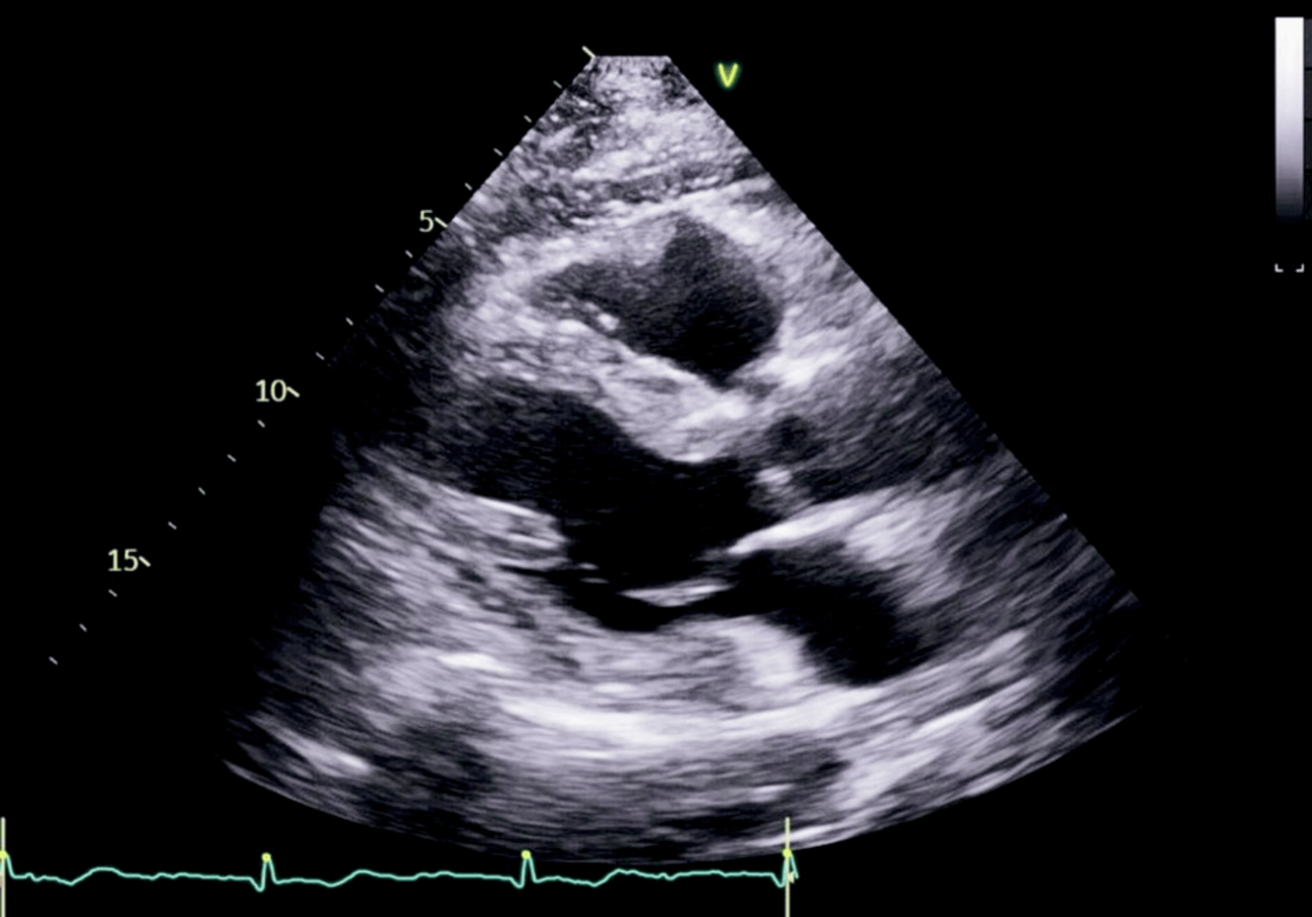
Parasternal long-axis view acquired via transthoracic echocardiography, visualizing the patient's cardiac anatomy

**Figure 3:**
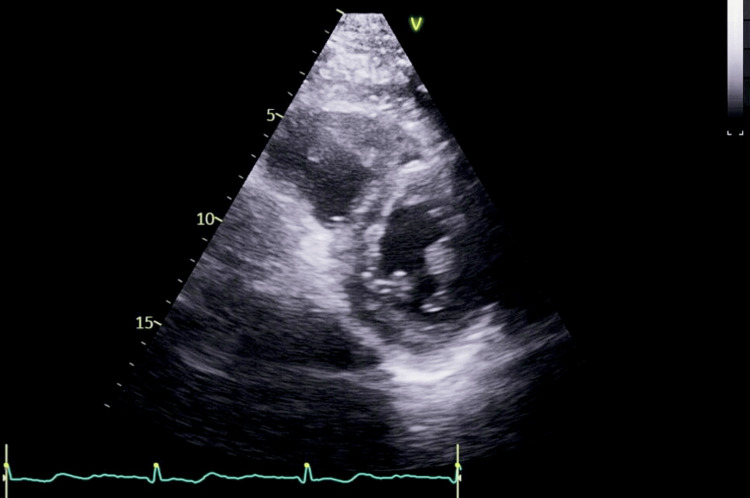
Parasternal short-axis view acquired via transthoracic echocardiography, visualizing the patient's cardiac anatomy

A 72-year-old man (119 kg, body mass index (BMI) 37 kg/m², American Society of Anesthesiologists (ASA) IV physical status classification) presented for excision of a right olecranon bursa due to chronic bursitis (Figure 1). His medical history included compensated alcoholic cirrhosis (Child-Pugh Class A, Model for End-Stage Liver Disease (MELD) 3.0, Model for End-Stage Liver Disease-Sodium (MELD-Na) 15) complicated by portal hypertension, gastrointestinal bleeding, non-bleeding esophageal varices, gastric antral vascular ectasia, and thrombocytopenia. Additional comorbidities included moderate pulmonary hypertension, obstructive sleep apnea, stage 3b chronic kidney disease, hypertension, type 2 diabetes mellitus, and chronic anemia. The patient also had a self-reported history of delayed emergence following general anesthesia, but prior anesthesia records were unavailable for review. Preoperative labs revealed hemoglobin 12.6 g/dL, platelet count 40×10^9^/L, creatinine 2.05 mg/dL, and international normalized ratio (INR) 1.0 (Table 1). His laboratory values remained stable for several months, with no reported bleeding or coagulopathy episodes. Transthoracic echocardiography (TTE) showed concentric left ventricular remodeling with preserved ejection fraction (61%), no regional wall motion abnormalities, and borderline right ventricular enlargement with normal function (Figures 2-3). The estimated right ventricular systolic pressure was 32 mmHg. No intrapulmonary shunting was observed.

**Table 1:** Pertinent preoperative laboratory findings of the patient INR: international normalized ratio; g: gram; dL: deciliter; L: liter; mg: milligram

Blood parameter	Laboratory value of the patient	Reference range (units)
Hemoglobin	12.6	13.2-16.6 (g/dL)
Platelet count	40	135-317 (×10^9^/L)
Creatinine	2.05	0.74-1.35 (mg/dL)
INR	1.0	0.9-1.1

**Figure 4:**
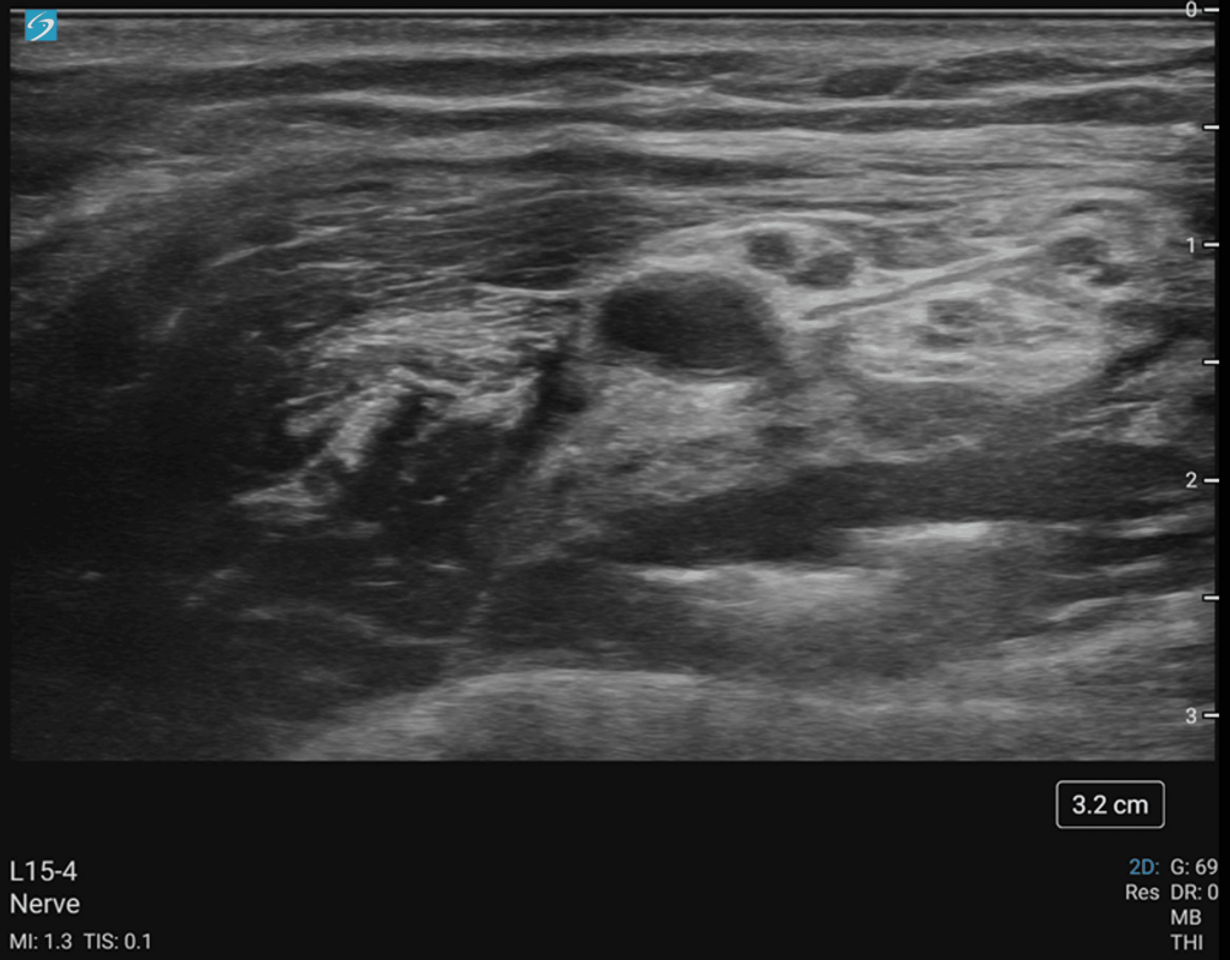
Ultrasound-guided axillary nerve block on the patient with a 15-4 MHz linear transducer and a 20-gauge, 4-inch echogenic B. Braun Ultraplex 360® needle

**Figure 5:**
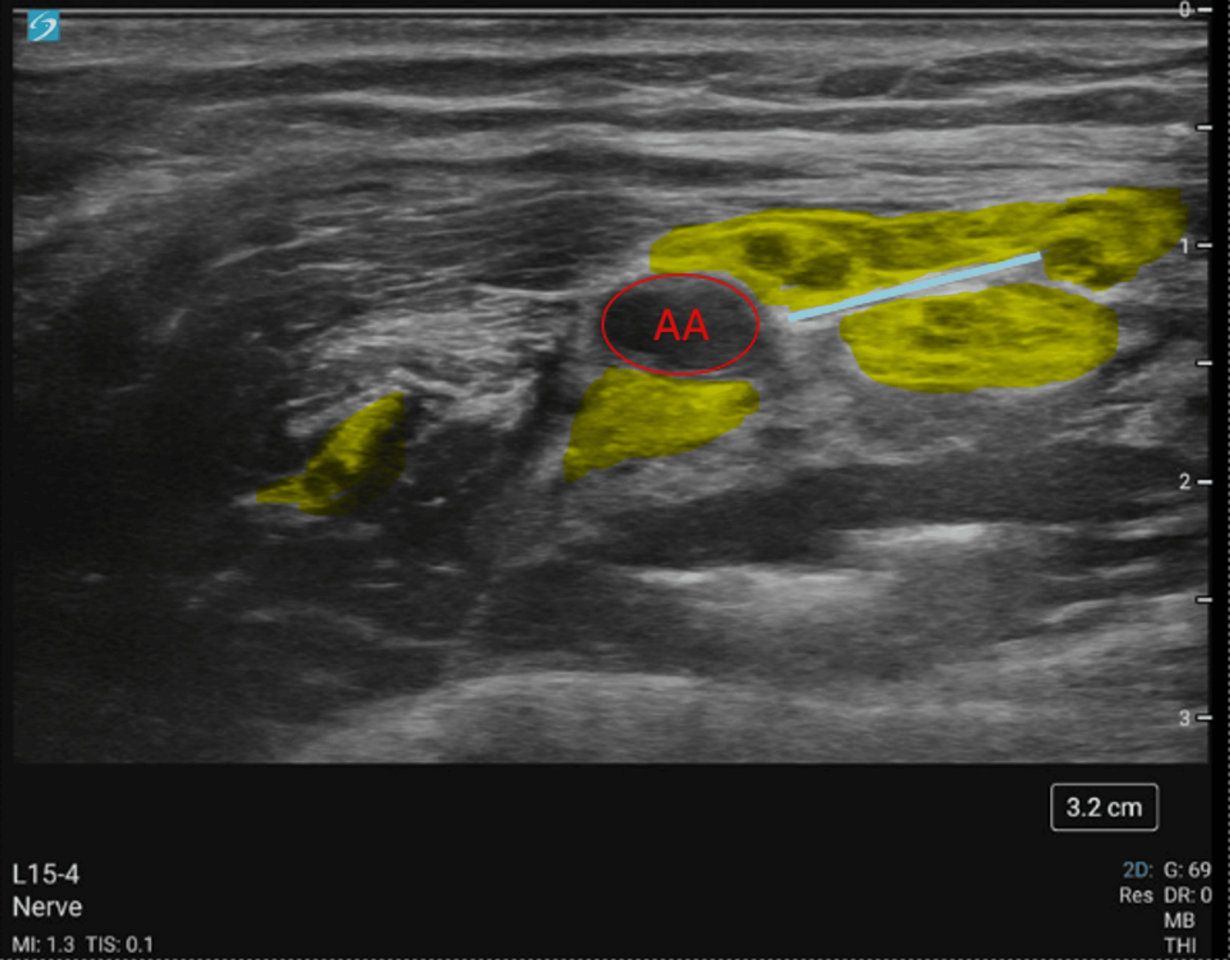
Labeled ultrasound-guided axillary nerve block on the patient with a 15-4 MHz linear transducer and a 20-gauge, 4-inch echogenic B. Braun Ultraplex 360® needle. The axillary vein was compressed in the ultrasound snapshot The red circle highlights the axillary artery (AA). Yellow areas highlight the brachial plexus. The blue rectangle highlights the collapsed axillary vein.

Given the patient's comorbidities and prior delayed emergence from general anesthesia, a regional technique was selected to minimize systemic anesthetic exposure. In addition, regional anesthesia helped avoid intubation and positive pressure ventilation with its associated risk of bronchospasm; this reduced the risk of acute right ventricular failure in a patient with pulmonary hypertension. An axillary brachial plexus block was performed using a 15-4 MHz linear transducer and a 20-gauge, 4-inch echogenic B. Braun Ultraplex 360® needle (Melsungen, Germany). With the patient supine, the arm was abducted to 90°, externally rotated, and flexed. Under real-time ultrasound guidance, the needle was advanced, and the circumferential spread of local anesthetic was confirmed following negative aspiration. A total of 30 mL of 0.5% bupivacaine with 1:200,000 epinephrine was administered to block the radial, median, ulnar, and musculocutaneous nerves (Figures 4-5).

Post-block, the patient demonstrated appropriate sensory and motor blockade in the radial, median, and ulnar nerve distributions. Despite expected sensory loss in the lateral forearm, elbow flexion was preserved, suggesting incomplete blockade of the musculocutaneous nerve. No complications, such as hematoma, were observed.

The patient received monitored anesthesia care with a propofol infusion (35 mcg/kg/min), bolus doses of intravenous dexmedetomidine (20 mcg total), and remimazolam (10 mg total). Due to persistent biceps flexion, the surgeon administered 100 mg of ropivacaine around the bursa at the start of the procedure. Hemodynamic support included phenylephrine and glycopyrrolate. The procedure lasted 128 minutes with an estimated blood loss of 100 mL.

In the post-anesthesia care unit, the patient reported a pain score of 0/10, maintained appropriate sensory and motor blockade, and required no additional analgesia. He was discharged home the same day without complications.

## Discussion

Cirrhosis affects an estimated 112 million individuals globally, with rising prevalence [11]. Ziser et al. reported a 30-day perioperative mortality rate exceeding 10% and a complication rate of 30% in cirrhotic patients undergoing surgery under various anesthetic modalities [5]. Several of these risk factors, including male sex, elevated ASA status, and elevated creatinine, were present in our patient, underscoring the need for a tailored anesthetic approach.

Given the patient's comorbidities and anesthetic history, regional anesthesia was deemed preferable to general anesthesia. The nerve block would reduce the amount of opioids required, which was desirable in this patient with obstructive sleep apnea and pulmonary hypertension. The patient also had a documented history of delayed emergence following general anesthesia, raising concern for postoperative altered mental status and prolonged recovery. Regional anesthesia was selected to minimize the use of systemic agents that could exacerbate these risks. It also reduced the risk of hemodynamic instability during induction and prevented the adverse effects of positive pressure ventilation on right heart function associated with general anesthesia. Avoiding deep sedation also minimized the risk of hypercarbia, which could increase pulmonary vascular resistance, lead to acute right ventricular strain, and ultimately compromise cardiac output [9]. 

Regional anesthesia also offered the advantage of greater hemodynamic stability, which is particularly important in cirrhotic patients who typically exhibit baseline reductions in systemic vascular resistance, elevated cardiac output, and impaired autoregulatory capacity. Compared to general anesthesia, regional techniques in orthopedic surgeries reduce the need for medications requiring hepatic metabolism, provide superior perioperative analgesia, and are associated with a lower incidence of adverse drug effects, including postoperative delirium, and expedited hospital discharge [12].

Despite general recommendations favoring regional anesthesia in patients with liver disease, the literature remains limited regarding its use in those with cirrhosis complicated by coagulopathy or thrombocytopenia [13]. Accurately assessing bleeding risk in this population is inherently challenging due to the dynamic imbalance between procoagulant and anticoagulant factors [10,14]. Standard coagulation assays, such as INR and platelet count, may not reliably reflect true hemostatic function in cirrhotic patients [10,14-16]. Viscoelastic testing modalities such as thromboelastography (TEG) and rotational thromboelastometry (ROTEM) offer more accurate, real-time assessments of coagulation status and are increasingly favored in guiding transfusion strategies and intraoperative management, though availability may be limited [14].

An axillary brachial plexus block was ultimately selected to mitigate the risk of phrenic nerve palsy and pneumothorax, particularly in the context of underlying pulmonary hypertension [17]. The axillary approach was further favored due to its compressible location, which offers a safety advantage in the event of bleeding. The potential for local anesthetic systemic toxicity (LAST) was carefully considered, as patients with cirrhosis often exhibit reduced plasma protein binding and impaired hepatic metabolism of amide local anesthetics. Amide local anesthetics were preferred due to their longer duration of action, which was more suitable for the procedure. Moreover, although ester local anesthetics are metabolized independently of hepatic function, they are more commonly associated with allergic reactions compared to amide local anesthetics [18]. The patient received 250 mg of local anesthetic (100 mg ropivacaine and 150 mg bupivacaine) with epinephrine, remaining below the maximum recommended dose of 3 mg/kg. However, severe chronic liver disease may reduce the maximum safe dose of local anesthetic, although specific threshold values have not been clearly established. Notably, more than two hours elapsed between the administration of the two local anesthetics. The patient exhibited no clinical signs of LAST.

Interestingly, a sensory block was achieved in the distribution of the musculocutaneous nerve without complete motor blockade, suggesting a partial block of this nerve. Close collaboration with the surgical team enabled supplemental wound infiltration, which likely contributed to the avoidance of conversion to general anesthesia. The surgeon used ropivacaine to minimize the cardiotoxicity of the local anesthetic, as bupivacaine had already been administered. The use of peripheral nerve stimulation in conjunction with ultrasound guidance for musculocutaneous nerve identification may enhance the reliability of the block and can be considered in future cases [19].

This case demonstrates that, with careful technique selection and risk assessment, regional anesthesia can be safely employed in cirrhotic patients with coagulopathy. However, further research is needed to better define safety thresholds and outcomes in this population.

## Conclusions

Cirrhotic patients present unique anesthetic challenges due to multisystem involvement and altered coagulation. In this case, an axillary brachial plexus block provided a safe and effective alternative to general anesthesia, minimizing systemic drug exposure and supporting hemodynamic stability. This technique facilitated an uncomplicated and rapid recovery. Despite general support for regional anesthesia in cirrhosis, further studies are needed to guide its use in patients with significant coagulopathy.
